# Curcumin rescues *Caenorhabditis elegans* from a *Burkholderia pseudomallei* infection

**DOI:** 10.3389/fmicb.2015.00290

**Published:** 2015-04-10

**Authors:** Su-Anne Eng, Sheila Nathan

**Affiliations:** Faculty of Science and Technology, School of Biosciences and Biotechnology, National University of MalaysiaBangi, Malaysia

**Keywords:** *Burkholderia pseudomallei*, *Caenorhabditis elegans*, curcumin, antibiotic resistance, bacterial attenuation

## Abstract

The tropical pathogen *Burkholderia pseudomallei* requires long-term parenteral antimicrobial treatment to eradicate the pathogen from an infected patient. However, the development of antibiotic resistance is emerging as a threat to this form of treatment. To meet the need for alternative therapeutics, we proposed a screen of natural products for compounds that do not kill the pathogen, but in turn, abrogate bacterial virulence. We suggest that the use of molecules or compounds that are non-bactericidal (bacteriostatic) will reduce or abolish the development of resistance by the pathogen. In this study, we adopted the established *Caenorhabditis elegans*-*B. pseudomallei* infection model to screen a collection of natural products for any that are able to extend the survival of *B. pseudomallei* infected worms. Of the 42 natural products screened, only curcumin significantly improved worm survival following infection whilst not affecting bacterial growth. This suggested that curcumin promoted *B. pseudomallei*-infected worm survival independent of pathogen killing. To validate that the protective effect of curcumin was directed toward the pathogen, bacteria were treated with curcumin prior to infection. Worms fed with curcumin-treated bacteria survived with a significantly extended mean-time-to-death (*p* < 0.0001) compared to the untreated control. In *in vitro* assays, curcumin reduced the activity of known virulence factors (lipase and protease) and biofilm formation. To determine if other bacterial genes were also regulated in the presence of curcumin, a genome-wide transcriptome analysis was performed on curcumin-treated pathogen. A number of genes involved in iron acquisition and transport as well as genes encoding hypothetical proteins were induced in the presence of curcumin. Thus, we propose that curcumin may attenuate *B. pseudomallei* by modulating the expression of a number of bacterial proteins including lipase and protease as well as biofilm formation whilst concomitantly regulating iron transport and other proteins of unknown function.

## Introduction

*Burkholderia* pseudomallei is the causative agent of the tropical disease, melioidosis, which is endemic in Southeast Asia and northern Australia (Wiersinga et al., 2006). Melioidosis accounts for the high overall mortality in northeast Thailand and is reported to be the third leading cause of death from infectious diseases after human immunodeficiency virus (HIV)-AIDS and tuberculosis (Limmathurosatkul et al., 2010). Despite growing efforts to develop immunoprophylaxis treatment for melioidosis (Hara et al., 2009; Patel et al., 2011; Choh et al., 2013; Silva and Dow, 2013), currently there is no vaccine available for clinical use. Thus, treatment of melioidosis is still solely dependent on a lengthy course of antibiotics. Nevertheless, B. pseudomallei is becoming increasingly resistant to a diverse group of antimicrobials including third generation cephalosporins whilst quinolones and aminoglycosides have no reliable effect (Puthucheary and Sam, 2012). For example, two important antibiotic candidates in melioidosis treatment are ceftazidime and amoxicillin-clavulanic acid and cases of resistance have been reported in recent years (Wuthiekanun et al., 2011). Therefore, therapeutic options are limited and the difficulty in achieving sterilization is not fully understood. Hence, there is an urgent need for new alternative treatments for this disease.

To alleviate the worldwide problem of multidrug resistant (MDR) bacteria such as the carbapenem-resistant Enterobacteriacea (Kumarasamy et al., 2010; Zilberberg and Shorr, 2013), new strategies have been developed to reduce the impact of antibiotic resistance. As the search for new antibiotics is proving to be a major problem, an alternative strategy being pursued globally is the search for new anti-infectives. Unlike traditional antibiotics that are either bacteriostatic or bactericidal, anti-infectives are known to target bacterial virulence (Clatworthy et al., 2007; Papa et al., 2013; Sarabhai et al., 2013) or alternatively, improve host immunity (Hamill et al., 2008; Nijnik, 2013; Kong et al., 2014b). Inhibition of virulence should prevent the onset of infection, persistence and damage to the host without threatening bacterial survival, thus reducing selection pressure toward the development of antibiotic resistance (Clatworthy et al., 2007; Cegelski et al., 2008).

The versatile nematode, *Caenorhabditis elegans*, was selected as the host model for this study. *C. elegans* has been widely used as a tool to elucidate host-pathogen interactions (Tan et al., 1999; Garsin et al., 2001; Lee et al., 2011). Many of the bacterial virulence factors identified to be crucial in the killing of *C. elegans* were also required for pathogenesis in mammals hence promoting the importance of the worm as a tool in anti-virulence drug discovery (Moy et al., 2006; Adonizio et al., 2008). An important resource of molecules in the search for new drugs against human diseases is natural products (Newman and Cragg, 2012). The availability of these natural products and their derivatives has enabled the identification of potential candidate anti-infectives toward clinical pathogens using the *C. elegans* infection model platform (Moy et al., 2006; Kong et al., 2014a). In addition, the *in vivo* whole animal screen allows simultaneous assessment of drug pharmacokinetics and toxicity.

In this study, we utilized the established *C. elegans* - *B. pseudomallei* infection model (O'Quinn et al., 2001; Gan et al., 2002; Lee et al., 2013) as a screening tool to assess a collection of natural products as potential anti-infectives toward *B. pseudomallei*. This screen identified a natural compound, curcumin, as a positive hit based on its ability to prolong the lifespan of infected worms. Curcumin is a polyphenol extracted from *Curcuma longa*. It is commonly used in cuisine and folk medicine especially in South and Southeast Asian countries. Anecdotal evidence has proposed that curcumin exhibits a number of beneficial properties such as antioxidant, anti-inflammatory, antiviral, antibacterial, antifungal and anti-cancer activities (Aggarwal et al., 2007). Curcumin is known to be antibacterial toward various pathogens (Moghadamtousi et al., 2014) and this could be attributed to the iron chelating property of curcumin that impedes bacterial growth. However, in this study we observed continued survival and growth of *B. pseudomallei* in the presence of curcumin. Nevertheless, pretreatment of the bacteria with curcumin prior to nematode infection resulted in enhanced survival of infected worms proposing the selective action of curcumin on pathogen virulence. Further transcriptome analysis was undertaken to provide more insight into the possible mode adopted by curcumin in its role as a potential anti-infective agent against *B. pseudomallei*.

## Materials and methods

### Bacterial isolates and nematode strains

*B. pseudomallei* clinical isolates R15 (Lee et al., 2007), K96243 (Holden et al., 2004) and UM6 as well as *Enterococcus faecalis* V583 were grown in BHI broth, *Pseudomonas aeruginosa* strain PA14 in King's B broth, *Staphylococcus aureus* strain NCTC83254 and methicillin-resistant *S. aureus* (MRSA) strain ATCC33591 in Trypticase Soy (TS) media while *Escherichia coli* strain OP50 and *Salmonella typhimurium* SL1344 were cultured in Luria Bertani (LB) broth. All bacterial cultures were grown aerobically at 37°C overnight. The wild type *C. elegans* Bristol N2 (N2) and *rrf-3(pk1426);glp-4(bn2)* strains were obtained from the Tan Laboratory at Stanford University USA. The worms were maintained on nematode growth medium (NGM) agar plates seeded with a lawn of the standard laboratory food source, *E. coli* OP50. Age-synchronization of the worms was conducted by the hypochlorite treatment of gravid worms as described by Shapira and Tan (2008). N2 worms were made sterile through RNAi knockdown of the *pos-1* gene (Tabara et al., 1999) by feeding the worms with *E. coli* expressing double stranded RNA (dsRNA) directed against *pos-1*. Both sterile N2 and *rrf-3(pk1426);glp-4(bn2)* worms were grown at 25°C until they reached the young adult stage before they were used for infection.

### Natural products

A total of 42 natural products extracted from various plants and marine extracts were used in this study. These products were either obtained commercially or supplied by the Institute for Pharmaceuticals and Neutraceuticals Malaysia (IPharm). Preparation and the origin of samples UE-01-1 to UE-20 are described in Kong et al. (2014a). Extracts UE-21 and UE-22 were purchased from Cosway Pte. Ltd while UE-23 and UE-24 were acquired from ONI Global Pte. Ltd. and Cambert Ptd. Ltd., respectively. Information on the 42 natural products is available in Supplementary Data 1. Stock solutions for all the natural products were prepared in dimethyl sulfoxide (DMSO), filtered with a 0.2 μm membrane filter and stored at −20°C until further use.

### Anti-infective screening

Screening of natural products was performed based on the protocol by Moy et al. (2006) with some modifications. Firstly, the screening medium comprising of 90% M9 buffer, 10 μg/mL cholesterol, 10% *B. pseudomallei* overnight culture and 200 μg/mL natural extract was prepared and aliquoted into individual wells (750 μL) in a 24-well plate. Each extract was tested in triplicate wells. Infection began when 10 *pos-1* treated N2 young adult worms were transferred into each well and the plate was incubated at 25°C without agitation. In control wells, *B. pseudomallei* was replaced with *E. coli* OP50 while the extract was replaced with 1% DMSO. Worm survival was scored manually throughout the assay. Worms were scored as dead when they did not respond to gentle probing with a platinum worm picker. An extract was considered a positive hit if it resulted in an average worm survival of >50% in at least 2 out of 3 independent assays at a point when 50% of the untreated worms survived the infection.

### Determination of antimicrobial properties of positive hits

#### Disc diffusion test

The disc diffusion assay was carried out according to the method outlined by the Clinical and Laboratory Standards Institute (CLSI) M2-A9 (CLSI, 2006). The inoculum was prepared by diluting a *B. pseudomallei* overnight culture to OD_595_ = 0.5 (10^8^ cfu/mL). A total of 100 μL of the inoculum was spread on Mueller Hinton agar using glass beads. Each positive hit (20 mg/mL) was impregnated into individual sterile filter paper discs (Whatman No. 1, 5 mm) and left to dry overnight. Tetracycline (200 μg/mL) was used as the positive control while DMSO was used as the negative control. Dried impregnated discs were placed on the *B. pseudomallei* lawn and incubated at 37°C for 20 h. The formation of a clear zone of inhibition around the disc indicated the presence of antimicrobial activity for the extract.

#### Broth microdilution antimicrobial test

The observed antimicrobial property of positive hits was confirmed through the broth microdilution minimum inhibitory concentration (MIC) test (Wiegand et al., 2008). Positive hits were diluted twofold from 500 to 0.24 μg/mL in a 96-well plate and an inoculum of 10^6^ cfu/mL *B. pseudomallei* was added into each well. This test was conducted using the anti-infective screening medium (90% M9 buffer + 10% BHI broth) in order to mimic the assay conditions above. The plate was incubated at 37°C for 20 h. The same concentrations of tetracycline and DMSO were included as the positive and negative controls, respectively. The MIC of a positive hit was defined as the lowest concentration where no visible bacterial growth was observed. The minimum bactericidal concentration (MBC) was determined by spreading the culture from each well onto agar plates. The lowest concentration with no bacterial growth on an agar plate was deemed the MBC value for the particular hit.

Determination of curcumin antimicrobial effects on *B. pseudomallei* was conducted in standard medium (BHI broth) similar to the broth microdilution method above. Concentrations of curcumin ranging from 0.6 to 1200 μM were tested along with their vehicle DMSO and tetracycline as controls. After incubation, cultures from each concentration were serially diluted, spotted on agar, incubated and the bacterial colony forming units (CFU) were enumerated. However, for the effect of curcumin on *P. aeruginosa*, *E. coli* OP50, *S. typhimurium*, *E. faecalis*, *S. aureus* and MRSA, CFU was not counted. Instead, cultures were spotted directly onto agar plates and incubated. The bacterial growth in each spot was visually compared to their respective DMSO control. The bacteria were considered susceptible to curcumin if there was a decrease in the visible growth when compared to the vehicle DMSO.

### *C. elegans* survival assay

The liquid based survival assay was performed in the same manner as the anti-infective screen but with minor modifications. The *pos-1* treated N2 young adult worms were replaced with 30 sterile *rrf-3(pk1426);glp-4(bn2)* young adult worms. The *rrf-3(pk1426);glp-4(bn2)* worms carry a *glp-4* temperature sensitive mutation which results in germline-deficient worms when grown at 25°C (Beanan and Strome, 1992). Therefore, *rrf-3(pk1426);glp-4(bn2)* worms were maintained at 16°C to produce gravid worms and subjected to hypochlorite treatment to obtain eggs. The eggs were seeded on NGM agar and grown to sterile young adult worms at 25°C that were then used for the infection assay. Alive and dead worms were scored every 4 h post-infection. Each extract was assayed in triplicate wells corresponding to a total of 90 worms. In control wells, extract was replaced with DMSO whereas *B. pseudomallei* was replaced with *E. coli* OP50.

We used a modified survival assay to investigate the effect of curcumin on the pathogenicity of *B. pseudomallei* in *C. elegans*. Firstly, *B. pseudomallei* was cultured overnight in the presence of curcumin (50 μM, 100 μM or 543 μM) at 37°C. The curcumin-treated cultures were then centrifuged at 4000 g for 10 min at 4°C. The supernatant was removed and the bacterial cell pellet was resuspended with fresh BHIB broth. The washing step was repeated to minimize curcumin content. The cell suspension was then used in the preparation of assay medium, which comprised only of 90% M9 buffer, 10% curcumin-treated culture and 10 μg/mL cholesterol without any further addition of curcumin into the assay medium. Infection began when 30 sterile *rrf-3(pk1426);glp-4(bn2)* young adult worms were added into the wells. For the negative control, *B. pseudomallei* was treated with DMSO instead of curcumin.

### Colony forming unit (CFU) assay

*In vivo* bacterial counts were determined according to the method of Ooi et al. (2012). At each time point, 10 live worms infected with 50 μM curcumin-treated bacteria were randomly picked and briefly anesthetized in 25 mM Lev. The worms were washed twice in 200 μl antibiotic cocktail comprising 25 mM Lev and 500 μg/ml kanamycin followed by incubation for at least 45 min to completely kill bacterial cells associated with the worm cuticle. Next, the worms were washed three times with 200 μl of 25 mM Lev to eliminate the killed bacteria and residual antibiotic. During the last washing step, the Lev was removed, leaving about 5 μl in the tube. Prior to adding 50 μl 1% Triton X (Sigma-Aldrich, X100), the worms were enumerated and then homogenized with a motorized pestle. Serial dilutions were performed on the worm lysates. Briefly, 10 μl of the worm lysate was spotted on Ashdown agar supplemented with 100 μg/ml Cm using the drop plate method with modifications. Colonies were counted after incubating the plates at 37°C for 48 h. Average colony numbers obtained from visually separate colonies were used for statistical analysis. Bacterial CFU per worm was calculated using the formula: (average colony number × dilution factor × 55 μl worm lysate)/(10 μl worm lysates plated × number of worms). Three independent replicates were performed for the experiment. The phrase “55 μl worm lysate” (50 μl 1% Triton X + 5 μl Lev containing the worms) refers to the total worm lysate of which 10 μl was used for plating.

### The effect of curcumin and ethylene glycol tetraacetic acid (EGTA) on *B. pseudomallei* replication

An overnight *B. pseudomallei* culture was diluted 100 fold in fresh broth supplemented with curcumin (50 μM, 100 μM or 543 μM) or EGTA (5mM or 10 mM). The subculture was thereafter incubated shaking (250 rpm) at 37°C. At 2-hourly intervals, CFU counts were determined and a growth curve was plotted. The doubling time (g), was calculated from the exponential phase of the growth curve using the formula: *g* = t log 2/(log N*_t_* – log N_0_) where *N*_0_ = number of CFU at a point during log phase, *N_t_* = number of CFU at a different time point during log phase and *t* = time interval between *N*_0_ and *N_t_*. DMSO (0.1%) was used as the control.

### The effect of curcumin on *B. pseudomallei* virulence factors

#### Hemolysin test

To determine if curcumin affects *B. pseudomallei* hemolysin, curcumin-treated (50 μM, 100 μM or 543 μM) *B. pseudomallei* overnight culture was streaked on 5% rabbit blood agar and incubated at 37°C for 24 h. During the preparation of the agar, the same amount of curcumin was added while the agar was still in molten state. As a control, *B. pseudomallei* was cultured in the presence of DMSO (0.1%, 0.2% or 1.0%) and streaked on DMSO supplemented blood agar. After 24 h incubation, the presence and type of hemolysis was recorded.

#### Lipase test

The effect of curcumin on *B. pseudomallei* lipase was determined using Rhodamine B-olive oil agar (Kouker and Jaeger, 1987). *B. pseudomallei* was co-cultured with 50 μM, 100 μM or 543 μM curcumin overnight and subsequently spotted on Rhodamine B-olive oil agar supplemented with the same concentration of curcumin and then incubated at 37°C for 48 h. As a control, *B. pseudomallei* was cultured in the presence of DMSO (0.1%, 0.2% or 1.0%) and streaked on DMSO supplemented Rhodamine B-olive oil agar. After 48 h, the plates were observed under UV light (350 nm). Lipase activity was indicated by orange-red fluorescent colonies.

#### Protease test

The effect of 50 μM curcumin on *B. pseudomallei* protease was determined using 3% skim milk agar (Oxoid, UK). The overnight curcumin-treated bacterial culture was spotted on skim milk agar supplemented with 50 μM curcumin and incubated at 37°C for 48 h. As a control, *B. pseudomallei* was cultured with 0.1% DMSO and spotted on 0.1% DMSO supplemented skim milk agar. After 48 h incubation, the formation of a halo was observed.

### The effect of curcumin on *B. pseudomallei* biofilm production

The biofilm assay was conducted according to the protocol of Koh et al. (2013). Firstly, the known high biofilm producer *B. pseudomallei* UM6 was cultured overnight with 50 μM curcumin at 37°C. The overnight culture was diluted using fresh broth (BHI broth + 50 μM curcumin) to OD_595_ = 1 to obtain a standardized inoculum. For the negative control, 50 μM curcumin was replaced with 0.1% DMSO. The standardized inoculums (200 μL) were dispensed into 8 wells of a 96-well plate and incubated at 37°C for 48 h. Uninoculated BHI broth was included as the blank control. Following incubation, the wells were washed with 1X phosphate buffered saline (PBS) to remove non-adherent bacteria. Thereafter, the wells were fixed with 200 μL 99% (v/v) methanol for 15 min and air-dried at room temperature. The wells were then stained with 200 μL filtered 2% crystal violet for 5 min. The excess stain was washed with water and the wells were air-dried. The stain bound bacterial cells were solubilized with 200 μL of 95% (v/v) ethanol and the released stain was measured at 570 nm using a microplate reader (Sunrise, Tecan, Switzerland).

### Whole transcriptome analysis

The transcriptome analysis was conducted using the *B. pseudomallei* array and protocol previously described by Chieng et al. (2012). In brief, *B. pseudomallei* was cultured for 10 h (log-phase) with 50 μM curcumin or 0.1% DMSO as control at 37°C. Total RNA was extracted using Trizol, purified with Qiagen's RNeasy Mini Kit and on-column DNase I digestion was performed. The concentration, quality, and integrity of all RNA samples were analyzed using the Nanodrop® ND-1000 and Agilent 2100 Bioanalyser. Only RNA samples with a RIN value of ≥ 9 were selected for the next step. The RNA samples were polyadenylated using the A-plus™ Poly (A) Polymerase Tailing Kit (Epicentre). cDNA synthesis, labeling, and hybridization were performed according to the Agilent one-color microarray protocol. The polyadenylated RNA samples were reverse transcribed to cRNA, amplified, and labeled using Agilent's Low Input Quick Amp Labelling Kit. Next, the labeled cRNA samples were purified with the RNeasy Mini Kit and subjected to Nanodrop® ND-1000 reading. Only purified samples with a yield >1.65 μg and specific activity of >9.0 pmol Cy3 per μg cRNA were used for hybridization. The purified labeled cRNA samples (800 ng) were fragmented using the Agilent Fragmentation Buffer. This step was conducted at 60°C for 30 min. After that, the fragmentation reaction was stopped by cooling the samples on ice for 1 min followed by the addition of Agilent 2× Hi-RPM Hybridization Buffer. The hybridization samples were centrifuged at 13000 *g* for 1 min at room temperature. A total of 40 μL of each of the hybridized samples was loaded onto each array on the probe slide. The array was hybridized at 65°C for 17 h. Washing steps were done with the Agilent GE Wash Buffer according to the manufacturer's protocol. The arrays were then scanned on the Agilent Technologies Scanner.

Spot intensities and quality control features of each array were extracted using the Agilent Feature Extraction Software. The processed signals were filtered and normalized with BRB-ArrayTools (http://linus.nci.nih.gov). The resultant data were subjected to TIGR-MeV software for Significance Analysis of Microarray (SAM) analysis in which a two class unpaired analysis was performed and genes with a false discovery rate (FDR) < 0.01 and fold change ≥ 1.5 were defined as significantly differentially expressed. Functional classifications were carried out based on Comprehensive Microbial Resources (CMR) annotations (www.cmr.jcvi.org). Gene function enrichment analysis was performed against the DAVID 6.7 database (http://david.abcc.ncifcrf.gov/home.jsp) using the Fisher Exact test with Benjamini and Hochberg multiple testing correction (*p* < 0.05). The microarray result was validated using quantitative real-time PCR (qRT-PCR) analysis. qRT-PCR was conducted with the iScript One-Step RT-PCR kit with SYBR Green according to the manufacturer's instructions (Bio-Rad Laboratories) on the Bio-Rad iCycler. The gene BPSL2758 (*glyA*) that encodes for hydroxymethyltransferase was used as the reference as it did not show any significant changes in expression from the microarray experiment.

### Quantitative estimation of siderophore

The ability of curcumin to induce *B. pseudomallei* siderophore production was confirmed by using the Chrome Azurol S-liquid assay (CAS) (Schwyn and Neilands, 1987). The CAS assay detects a change in color from the blue CAS-iron complex to orange free dye when the bound iron is competitively chelated by a higher iron affinity siderophore from the complex. *B. pseudomallei* was cultured under the same conditions as that for the transcriptome experiment (10 h log phase) with either 50 μM curcumin or vehicle 0.1% DMSO at 37°C. The cultures were then centrifuged at 4000 *g* for 10 min at 4°C. The supernatants were filtered with a 0.2 μm membrane filter to remove residual bacterial cells. The supernatants (100 μL) were then transferred into 5 wells in a 96-well plate, representing 5 technical replicates. An equal amount of CAS assay solution (Schwyn and Neilands, 1987) containing 4 mM 5-sulfosalicylic acid was added into each well. The 5-sulfosalicylic acid acts as a CAS shuttle solution to increase the rate of iron exchange. Two reference blanks (BHI broth + 50 μM curcumin and BHI broth + 0.1% DMSO) without the addition of bacteria culture were included in the assay. Upon addition of the CAS assay solution, the plate was measured at 630 nm after equilibrium was reached (approximately 1 h). The relative percentage of siderophore produced by both untreated and curcumin-treated *B. pseudomallei* was then calculated and compared.

### Statistical analysis

Results from worm survival assays were analyzed using the Kaplan-Meier nonparametric survival analysis in Statview® 5.0.1 (SAS Institute, Inc). The data were presented as mean ± standard deviation (SD) of a representative from at least two independent experiments. The data from the rest of the assays were expressed as mean ± standard error of the mean (SE) from at least two independent assays. Statistical analyses were performed using the unpaired, two-tailed Student's *t*-test.

## Results

### Identification of potential anti-infectives using the *C. elegans - B. pseudomallei* system

**Figure 1:**
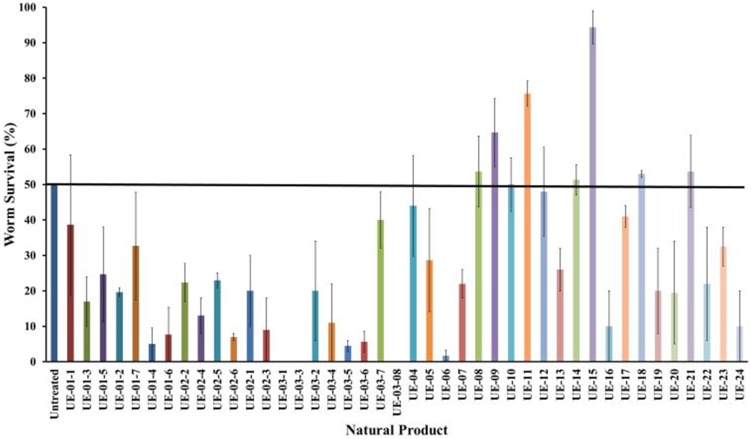
**Natural products enhanced the survival of *B. pseudomallei* infected worms**. The chart above compares the hits from the screen performed on 42 natural products (200 μg/mL). The data are plotted at the point where 50% of the untreated worms survived the infection. Natural products resulting in survival exceeding the untreated control were selected for further analysis. Results are expressed as mean ± SE from at least two independent assays.

Based on previous studies, *B. pseudomallei* is able to infect and kill *C. elegans* (O'Quinn et al., 2001; Gan et al., 2002; Lee et al., 2013). Using this system, several possible virulence determinants have been successfully elucidated (Gan et al., 2002; Puah et al., 2014). In this study, we extended the utility of the *B. pseudomallei* - *C. elegans* infection model to screen for anti-infectives that rescue the worm from infection. With this approach, we aimed to identify potential hits that attenuate the bacterial virulence factors. Throughout the study, the *B. pseudomallei* R15 clinical isolate was used unless stated otherwise. From the screen of 42 natural products, seven positive hits were obtained (Figure 1). These positive hits were UE-08, UE-09, UE-11, UE-14, UE-15, UE-18, and UE-21. These positive hits were able to enhance the survival of infected worms exceeding 50% survival at a point where half of the untreated worms succumbed to infection. We also noted that treatment with a number of these natural products resulted in lower worm survival when compared to the untreated control. Testing of selected natural products on *E. coli* OP50 fed worms indicated that these compounds are toxic to the host. For example, UE-03-4 and UE-03-5 treated worms killed the worms even in the absence of infection when compared to the untreated control (data not shown).

**Figure 2:**
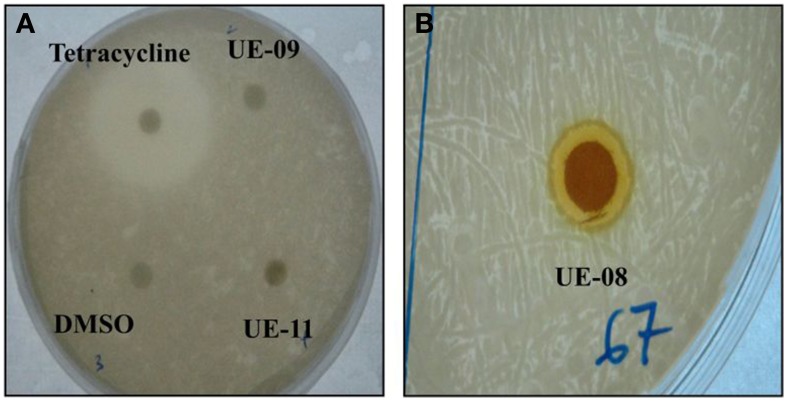
**Antimicrobial property of positive hits toward *B. pseudomallei***. **(A)** A clear zone of 30 mm was formed for the positive control (tetracycline) while no zone of inhibition was observed for the negative control (DMSO). No inhibition zones were formed for UE-09 and UE-11 implying that they did not exert antimicrobial effect on the bacteria. **(B)** The formation of a clear inhibition zone (9 mm) around the disc indicated susceptibility of the bacteria to UE-08.

The extended lifespan of infected worms in the presence of these positive hits could be attributed to the possible antimicrobial property of the natural products toward *B. pseudomallei*. However, we were only interested in potential extracts that were not antimicrobial in nature to reduce the possibility of the pathogen developing resistance to the selected hit(s). To delineate the selected positive hits into those with or without antimicrobial activity toward *B. pseudomallei*, we performed the disc diffusion and MIC tests. From the disc diffusion test, only one hit, UE-08, formed a zone of inhibition around the disc (Figure 2). The antimicrobial effect of all the positive hits was also determined through the broth microdilution MIC test. Again, only UE-08 exhibited an MIC value of 250 μg/mL and an MBC value of 500 μg/mL. This proposed that UE-08 enhanced the survival of infected worms by reducing the number of infecting bacteria. UE-08 is the crude extract of *Curcuma longa*. One of the well-established active ingredients of *C. longa* is curcumin and in this study, curcumin is represented by the positive hit UE-15. However, UE-15 did not show any anti-*B. pseudomallei* activity in both tests. The antimicrobial activity of the crude extract could be due to the existence of other components of *C. longa* such as diarylpentanoids, monoterpenes, sesquiterpenes, diterpenes, triterpenoids, alkaloid and sterols (Li et al., 2011). For example, terpenoids from *C. longa* were reported to possess antibacterial killing of various pathogens (Afzal et al., 2013). Thus, UE-08 was omitted from further analysis.

**Figure 3:**
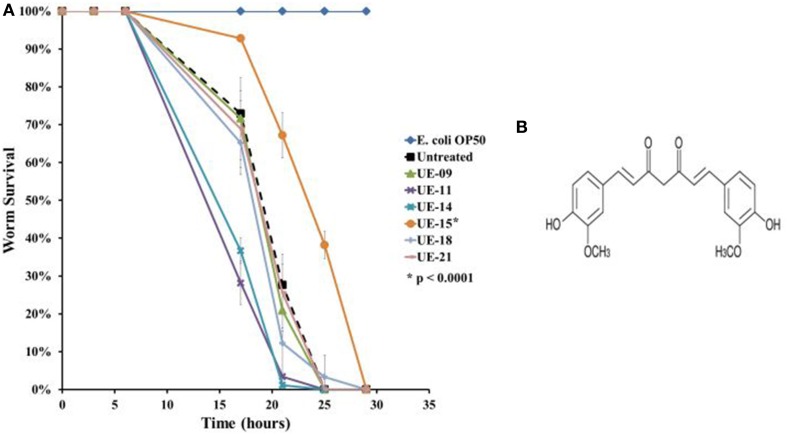
**UE-15 rescued *C. elegans* from *B. pseudomallei* infection**. **(A)** The survival curves for worms treated with the 200 μg/ml of the 6 positive hits. Treatment with UE-09, UE-11, UE-14, UE-18, and UE-21 failed to enhance the survival of infected worms whereas the addition of UE-15 extended the lifespan when compared to the untreated worms. Graph shows the mean ± SD from a representative of three independent assays. In a pair-wise analysis using the log-rank test, differences between UE-15 treated and untreated samples were significant (*p* < 0.0001). **(B)** Chemical structure of UE-15 or curcumin, a polyphenol from *C. longa*.

A more stringent survival assay was performed on the remaining positive hits utilizing a larger number of worms and additional time points to verify the anti-infective activities. Out of the positive hits tested, only UE-15 significantly prolonged the lifespan of *B. pseudomallei* infected worms (*p* < 0.0001) when compared to the untreated control (Figure 3A). The mean-time-to death (TD_mean_) of infected untreated worms was 21.019 ± 0.975 h and this was extended to 24.938 ± 0.522 h upon UE-15 treatment. As mentioned above, UE-15 is curcumin (Figure 3B), a polyphenol component of *C. longa*. Tables 1, 2 summarize the data obtained for the *C. elegans* survival assays and antimicrobial tests respectively.

**Table 1:** **The list of positive hits and their effects on the survival of *B. pseudomallei* infected *C. elegans***.

**Natural product ID/Treatment**	**Plant species/compound**	**Extraction solvent**	**Mean % of survival (primary screen)**	**Mean time to death (hours ± SD) (secondary screen)**
Untreated (+DMSO)			50.00	21.019 ± 0.332
UE-08	*C. longa*	Water	53.67	na
UE-09	*Centella asiatica*	Water	64.67	20.678 ± 0.301
UE-11	*Eurycoma longifolia*	Water	75.67	18.258 ± 0.227
UE-14	*Emblica officinalis*	Water	51.33	18.511 ± 0.215
UE-15	Curcumin		94.33	24.938 ± 0.522^*^
UE-18	*Silybum eburneum*	Water	53.00	20.202 ± 0.302
UE-21	Chorella		53.67	20.778 ± 0.318

*na-The survival of UE-08 treated worms was not available as this compound was not chosen for the secondary screen*.

* *p < 0.0001*.

**Table 2:** **The antimicrobial effect of positive hits against *B. pseudomallei***.

**Natural product ID/Treatment**	**Plant species/compound**	**Diameter of zone of inhibition (mm)**	**MIC (μg/mL)**	**MBC (μg/mL)**
Untreated (+DMSO)		–	–	–
Tetracycline		30	0.49	>500
UE-08	*C. longa*	9	250	500
UE-09	*C. asiatica*	–	–	
UE-11	*E. longifolia*	–	–	–
UE-14	*E. officinalis*	–	–	–
UE-15	Curcumin	–	–	–
UE-18	*S. eburneum*	–	–	–
UE-21	Chorella	–	–	–

*DMSO and tetracycline were used as negative and positive controls respectively*.

*- No visible reduction of growth was observed*.

### Curcumin rescues worms from *B. pseudomallei* infection with a mechanism distinct from conventional antibiotics

**Figure 4:**
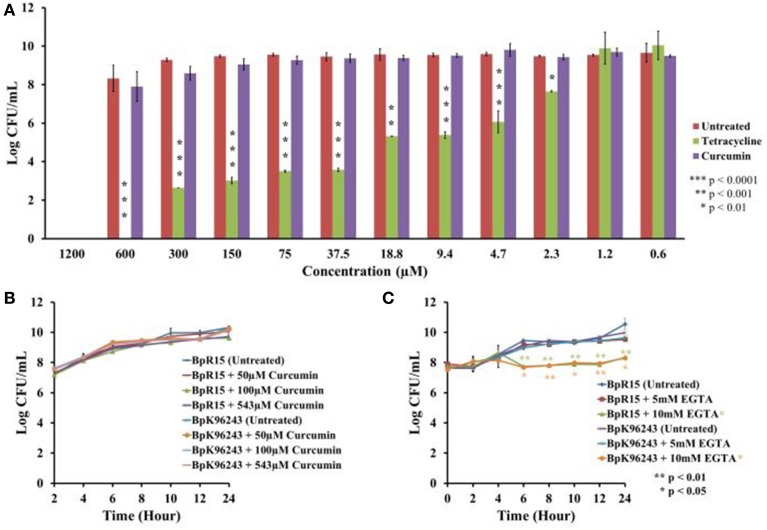
**Curcumin exhibited no antimicrobial effect on *B. pseudomallei*. (A)** At concentrations ranging from 0.6 μM to 600 μM, curcumin did not affect bacterial growth as reflected by the insignificant decrease in CFU when compared to the untreated control. DMSO at a concentration of 11.01% was antibacterial and prevented further analysis with curcumin at 1200 μM. The positive control, tetracycline, significantly decreased the number of CFU from 2.3 μM onwards. The bar chart corresponded to mean ± SE from three independent assays. **(B)** Growing *B. pseudomallei* in the presence of 50 μM, 100 μM, and 543 μM curcumin did not affect pathogen growth when compared to the untreated control. The graph shows the mean ± SE from three independent assays. **(C)** Treatment of *B*. *pseudomallei* with EGTA affected bacterial growth in a dose dependent manner. At 5 mM EGTA, no significant reduction of growth was observed. However, treatment with 10 mM EGTA significantly restricted the growth of *B. pseudomallei* when compared to the untreated control. The graph shows the mean ± SE from two independent assays.

From the screen, we identified curcumin as a potential anti-infective agent as it is capable of extending worm survival without compromising bacteria viability. Nevertheless, we performed further analysis to validate the observed antimicrobial effects. Bacteria were cultured in the presence of different concentration of curcumin and viable bacterial counts were enumerated. Curcumin at concentrations between 0.6 and 600 μM did not significantly reduce the number of CFU compared to the untreated bacteria (Figure 4A). At the higher concentration of 1200 μM, we are unable to make any conclusion as the concentration of DMSO (11.01%) used is itself antimicrobicidal (Ansel et al., 1969). Nevertheless, the data allowed us to conclude that 543 μM curcumin (the concentration used in the screens) was not antimicrobial toward *B. pseudomallei*. Tetracycline was used as the positive control and it significantly reduced (*p* < 0.01–*p* < 0.0001) the number of CFU at concentrations from 2.3 μM onwards (Figure 4A). The CFU data were used to plot growth curves for two strains of *B. pseudomallei*, K96243 and R15, grown in the presence of 50 μM, 100 μM or 543 μM curcumin. No significant difference in growth rates was observed when compared to the untreated bacteria (Figure 4B). The doubling times calculated for *B. pseudomallei* strain R15 cultured in increasing curcumin concentrations were 1.08 ± 0.07 h (50 μM), 1.15 ± 0.18 h (100 μM), and 1.26 ± 0.05 h (543 μM) compared to 1.09 ± 0.13 h for bacteria grown in the absence of curcumin. On the other hand, the doubling times for the reference strain K96243 were 1.20 ± 0.11 h (50 μM), 1.25 ± 0.04 h (100 μM), and 1.23 ± 0.06 h (543 μM) compared to 1.25 ± 0.08 h in the untreated bacteria. This confirmed that curcumin did not affect the growth of *B. pseudomallei*.

**Figure 5:**
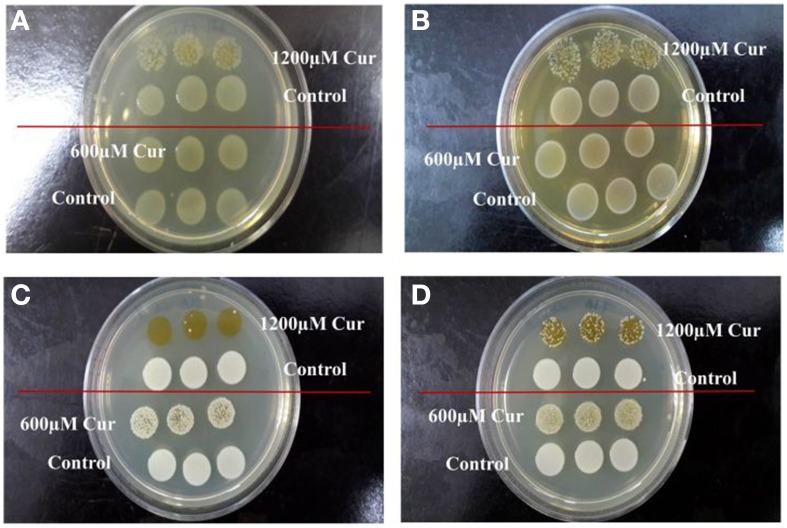
**Curcumin inhibited the growth of Gram negative and positive bacteria**. Antimicrobial effect of curcumin on **(A)** *E. coli* OP50, **(B)** *E. faecalis*, **(C)** *S. aureus*, and **(D)** MRSA. At 1200 μM curcumin, growth of **(A)** *E. coli* OP50 and **(B)** *E. faecalis* was reduced when compared to the untreated control. On the other hand, the growth of **(C)** *S. aureus* and **(D)** MRSA was inhibited by 600 μM curcumin and more prominent at 1200 μM.

To determine if curcumin demonstrated antimicrobial activity toward other pathogens, the antimicrobial assay was repeated on three Gram-negative bacteria (*E. coli* OP50, *P. aeruginosa*, and *S. typhimurium*) and three Gram-positive bacteria (*E. faecalis*, *S. aureus*, and MRSA). The growth of *E. coli* OP50 (Figure 5A) and *E. faecalis* (Figure 5B) were visibly reduced at 1200 μM curcumin. Furthermore, curcumin also affected the viability of both *S. aureus* (Figure 5C) and MRSA (Figure 5D) at 600 μM and 1200 μM curcumin. Our results are in concordance with the findings of Gunes et al. (2013) which reported the antibacterial effects of curcumin on *E. coli*, *E. faecalis*, *S. aureus*, and MRSA. Moreover, Kong et al. (2014a) also identified curcumin as a positive hit in a similar screen conducted on *S. aureus* in *C.elegans* infection model. However, in that report, the enhanced worm lifespan was an outcome of a reduced number of infecting bacteria in the presence of curcumin. Although our study has demonstrated a negative effect of curcumin on *P. aeruginosa*, the antimicrobial outcome of curcumin has been widely reported (Gunes et al., 2013; Moghadamtousi et al., 2014). In addition, the inability of curcumin to affect *S. typhimurium* growth *in vitro* mirrors the finding that curcumin promotes *S. typhimurium* proliferation (Marathe et al., 2010). Hence, we propose that, whilst in general curcumin is able to inhibit bacterial growth, it does not hinder *B. pseudomallei* growth. Thus, curcumin rescues *C. elegans* from a *B. pseudomallei* infection using a mechanism distinct from conventional antibiotics.

**Figure 6:**
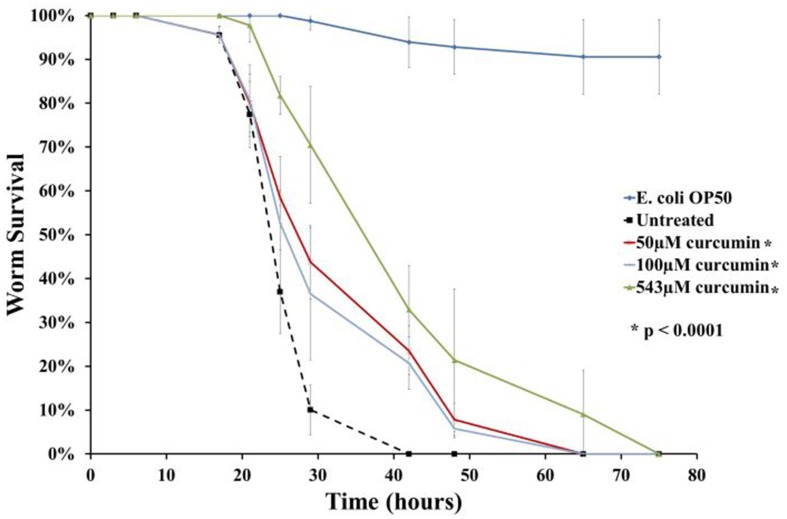
**Curcumin targets *B. pseudomallei* to rescue *C. elegans* from infection**. The chart above demonstrates worm survival following pre-treatment of *B. pseudomallei* with curcumin where the lifespan of worms was extended compared to the untreated control. The graph shows the mean ± SD from at least two independent assays. In a pair-wise analysis using the log-rank test, differences between treatment and control were significant.

As mentioned above, anti-infectives are able to either attenuate the pathogen or alternatively, strengthen host immunity. In both the screening and infection assays, curcumin was present in the growth medium. As such, we are unable to determine how the compound exerted its ability to rescue the infected worms. In this study, we were interested in pursuing anti-infectives that target bacterial virulence factors. To address this, the survival assay was modified whereby *B. pseudomallei* was pre-treated with 50 μM, 100 μM or 543 μM curcumin overnight prior to nematode infection. Following the overnight incubation, the curcumin-treated bacteria were pelleted, washed and resuspended in fresh BHIB broth. Curcumin pre-treated bacteria resulted in significant extension of worm survival when compared to worms infected with untreated bacteria (*p* < 0.0001) (Figure 6). The TD_mean_ was highest when the bacteria were treated with 543 μM curcumin: 43.910 ± 1.663 h compared to 26.724 ± 0.642 h for the untreated control. The TD_mean_ for both 50 μM and 100 μM were comparable at 34.839 ± 1.426 h and 33.194 ± 1.358 h, respectively. This suggested that curcumin is able to attenuate *B. pseudomallei* most likely by targeting its virulence factor(s). In addition, worms infected with 50 μM curcumin-treated bacteria were homogenized and bacterial counts were enumerated. Up to 12 h post infection, no significant difference was observed in bacterial intestinal colonization when compared to worms fed with untreated bacteria. The bacterial counts for worms infected with curcumin pre-treated bacteria at 4, 8, and 12 h post-infection were 1.58 ± 0.37, 1.26 ± 0.10, and 2.05 ± 0.26 log CFU/worm respectively whereas the bacterial counts for the untreated controls were 1.18 ± 0.03, 1.19 ± 0.01, and 1.97 ± 0.19 log CFU/worm respectively.

### Curcumin attenuates lipase, protease and biofilm formation

*B. pseudomallei* is known to secrete a number of virulence factors such as protease, lipase, hemolysin (Cheng and Currie, 2005) and the recently identified BLF-1 toxin (Cruz-Migoni et al., 2011). To evaluate the role of curcumin on selected secreted factors, we conducted *in vitro* biochemical tests on *B. pseudomallei* hemolysin, lipase and protease. The activity of these selected factors was monitored on blood agar, Rhodamine B-olive oil agar and skim milk agar, respectively. The respective agar plates were supplemented with curcumin to enable continuous exposure of the bacteria to curcumin throughout the assay period.

**Figure 7:**
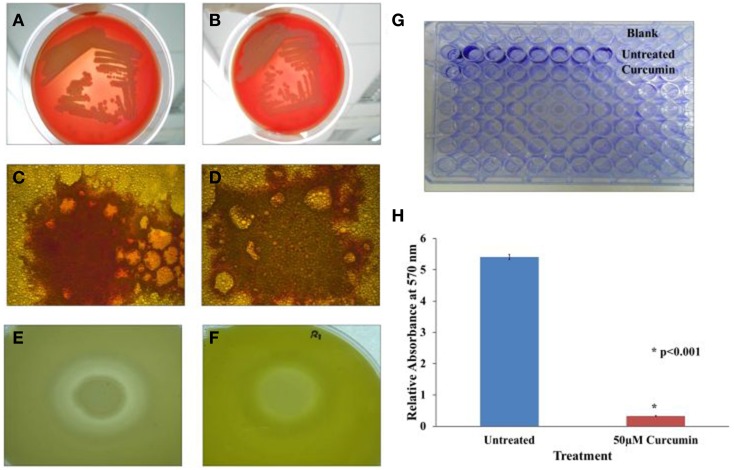
**Effect of curcumin on *B. pseudomallei* virulence factors**. In the hemolysin test, α hemolysis occurred in both **(A)** untreated and **(B)** curcumin-treated bacteria implying curcumin did not target *B. pseudomallei* hemolysin. In the lipase test, the presence of lipase is indicated by the presence of orange-red fluorescing colonies under UV light. The orange-red fluorescence was stronger in the untreated control **(C)** when compare to the curcumin-treated cells **(D)**. In the protease test, the formation of a large halo was observed in untreated cells **(E)** whilst a much smaller halo was observed in curcumin-treated bacteria **(F)**. Biofilm formation was also reduced following curcumin treatment **(G,H)**. The bar chart represents mean ± SD from two independent assays.

*B. pseudomallei* expresses the α and β classes of hemolysin. α-hemolysin is more commonly found among isolates and displays weak cytolytic activity that is observable only around heavy bacterial growth. On the other hand, β-hemolysin is produced infrequently but results in a clear hemolytic zone around single colonies (Ashdown and Koehler, 1990). *B. pseudomallei* R15 produced the more common α-hemolysin which partially lysed red blood cells (Figure 7A). This hallmark of α-hemolysis is indicated by the presence of green colored biliverdin, a by-product of hemoglobin breakdown. Treatment with curcumin did not affect the activity of hemolysin as α-hemolysis still occurred (Figure 7B).

In the lipase assay, we spotted untreated and curcumin-treated *B. pseudomallei* on Rhodamine B-olive oil agar (Kouker and Jaeger, 1987). Hydrolysis of olive oil by lipase produces free fatty acids. Interaction of free fatty acids with Rhodamine B produces orange-red fluorescence when viewed under UV light. Figures 7C,D depict our observations for both curcumin-untreated and curcumin-treated bacteria. Upon curcumin treatment, the intensity of the orange-red fluorescence was lower (Figure 7D) than the untreated control (Figure 7C). We also observed that this loss of intensity is more pronounced at higher concentrations of curcumin indicating that the effect of curcumin on lipase is dose dependent.

Curcumin also affected the activity or expression of *B. pseudomallei* protease. The principle of the skim milk agar assay is that protease will digest the opaque casein in the agar into transparent smaller peptides and amino acid resulting in the formation of a halo. The untreated *B. pseudomallei* culture hydrolysed the milk casein resulting in a clearly noticeable halo (Figure 7E). However, when the bacteria was grown in the presence of curcumin, the formed halo was smaller in size (Figure 7F). The ability of curcumin to inhibit protease activity has also been reported for curcumin-treated *P. aeruginosa* PA01 (Rudrappa and Bais, 2008).

Curcumin is also known to interfere with biofilm formation (Neelakantan et al., 2013; Packiavathy et al., 2014). Hence, we extended our investigation to determine if this was true for *B. pseudomallei* as well. As the R15 isolate is a poor biofilm producer, we used the *B. pseudomallei* UM6, a high biofilm producing strain (Chin et al., under review). In the presence of 50 μM curcumin, biofilm formation was significantly reduced (*p* < 0.001) when compared to the untreated control (Figures 7G,H).

### Transcriptome analysis revealed induction of genes involved in iron acquisition and transport as well as hypothetical proteins

The ability of curcumin to inhibit *B. pseudomallei* lipase and protease as well as biofilm formation hinted at the possibility that more virulence determinants could be targeted by curcumin. We therefore measured the global transcriptome expression levels of curcumin-treated *B. pseudomallei* and compared them to the expression of untreated bacteria. We treated with bacteria with 50 μM curcumin as this concentration was sufficient to rescue the infected worms (Figure 6) and also suppressed lipase and protease activity in addition to biofilm formation.

**Figure 8:**
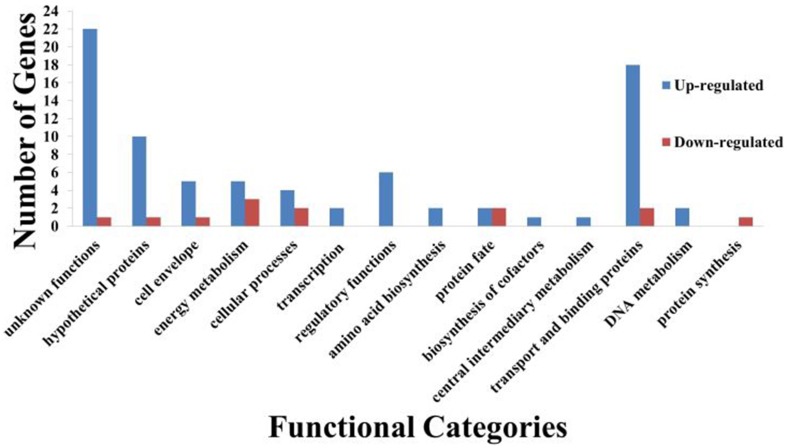
**Functional classification of curcumin regulated *B. pseudomallei* genes**. A total of 88 genes were differentially regulated by curcumin with 76 genes up-regulated whilst 12 down-regulated. Bars indicate number of genes in each group that were significantly regulated by curcumin. Genes were divided into functional categories based on Comprehensive Microbial Resources (CMR) annotations.

From the transcriptome analysis, we noted that curcumin significantly regulated the expression of 88 genes with 76 genes up-regulated and 12 genes down-regulated (Supplementary Data 2). Functional classification of these genes (Figure 8) showed that most of the induced genes were proteins of unknown function, hypothetical proteins and transport and binding proteins. Gene function enrichment analysis on the induced genes based on the KEGG biochemical pathways showed significant enrichment (*p* = 2.04 × 10^−5^) of ATP binding cassette (ABC) transporters specifically involving iron transport. On the other hand, no significant enrichment function was generated for the 12 repressed genes. Thus, all further discussion is limited to only the up-regulated genes.

Curcumin is a known iron chelator (Jiao et al., 2006), hence, it was not surprising to note the induction of genes involved in iron acquisition and transport (discussed below). A number of studies have proposed that the therapeutic effects of curcumin can be attributed to its iron chelating properties (Baum and Ng, 2004; Jiao et al., 2009). In our study, the addition of curcumin led to reduced iron concentration in the growth media as a result of the chelation. Our findings are similar to data presented by Tuanyok et al. (2005) and Alice et al. (2006) whereby iron transport related genes were up-regulated in iron-deficient growth conditions. Iron is essential for the growth and development of all pathogens. Under iron-restricted conditions, bacteria overexpressed genes responsible for iron uptake in order to maintain viability (Tuanyok et al., 2005).

In Gram negative bacteria, transport of iron from the environment into the cell is an active process that requires the TonB system (Krewulak and Vogel, 2008). In brief, Gram negative bacteria secrete siderophores, molecules with high affinity for iron, to scavenge iron from the environment. The TonB system drives a proton motive force from the cytoplasmic membrane to TonB dependent transporters (TBDTs) located at the outer membrane. The energized TBDTs then actively transfer iron bound siderophores into the cell (Krewulak and Vogel, 2008). The TonB system is made up of three components: TonB, ExbB, and ExbD. In *B. pseudomallei*, these proteins are encoded by BPSS0368, BPSS0367, and BPSS0366 respectively. From our analysis, all three genes were up-regulated following curcumin treatment.

As mentioned earlier, functional enrichment analysis revealed that the majority of the induced genes are ABC transporters. We mapped our list of up-regulated genes to the previously annotated *B. pseudomallei* ABC systems (Harland et al., 2007) and noted that five of the ABC systems involved in iron acquisition were induced. The identity of the induced genes (12) corresponding to each system is presented in Table 3.

**Table 3:** **Curcumin-induced ABC-related iron acquisition genes**.

**ABC System**	**Allocrite**	**Gene**	**Gene Description**
1	Malleobactin	BPSL1779	Putative siderophore biosynthesis related ABC transport protein
2	Pyochelin	BPSS0590	Probable ATP-binding component of ABC transporter
3	Ferric citrate or Ornibactin	BPSL1781	Extracytoplasmic-function sigma-70 factor PvdS
	Ferric citrate or Ornibactin	BPSL1783	Putative iron transport-related exported protein
	Ferric citrate or Ornibactin	BPSL1784	Putative iron transport-related ATP-binding protein
4	Heme	BPSS0240	Hemin ABC transport system, ATP-binding protein
	Heme	BPSS0241	Hemin ABC transport system, membrane protein
	Heme	BPSS0244	Exported heme receptor protein
5	Siderophore	BPSS1029	tonB-family receptor
	Siderophore	BPSS1204	Iron transport receptor protein
	Iron (III)	BPSL1277	Putative ABC transport system, membrane protein (possibly iron-related)
	Iron (III)	BPSL1278	Putative ABC transport system, iron-binding exported protein

**Figure 9:**
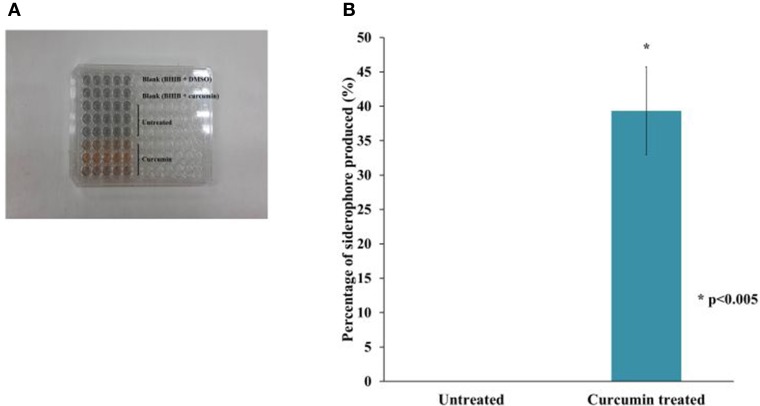
**The effect of curcumin on *B. pseudomallei* siderophore. (A)** The CAS solution changes color from blue to orange upon curcumin treatment whereas the mixture for untreated control remained blue. **(B)** The amount of siderophore detected was significantly higher in curcumin-treated bacteria than in the untreated control. The bar chart represents mean ± SE from two independent assays.

In addition to the TonB and ABC systems, we also observed an enrichment of siderophore-related genes. This suggests an increase in the biosynthesis of hydroxamate-type siderophores such as malleobactin and ornibactin as well as a mixed catecholate-hydroxamate pyoverdine corresponding to the over expressed BPSL1774-BPSL1787 cluster of genes. Within the BPSS0581–BPSS0594 cluster involved in pyochelin synthesis, a number of genes were also up-regulated, again, proposing the bacterial response to the low-iron environment in the presence of curcumin. We determined the amount of siderophore present in the growth media after curcumin treatment with the quantitative CAS-liquid assay. The amount of siderophore detected was significantly higher (*p* < 0.005) than the untreated control (Figure 9).

As iron is crucial for the optimal function of oxygen transport in living organisms, deprivation of iron will restrict cell growth and replication (Tuanyok et al., 2005). However, we had earlier noted that curcumin did not suppress the *in vitro* growth of *B. pseudomallei* (Figures 4A,B). On the other hand, in the presence of 5 mM EGTA, another known iron chelator, no apparent difference in growth rates was observed between the EGTA treated and untreated *B. pseudomallei* (Figure 4C). However, when the bacteria were treated with 10 mM EGTA, growth of both strains, K96243 and R15, was significantly reduced (*p* < 0.01–*p* < 0.05) from 6 h onwards when compared to the untreated control (Figure 4C). This is as expected as noted above, iron is essential for bacterial growth. For example, the addition of 2,2′-dipyridyl (DIP), an iron chelator, inhibited the growth of bacteria in a dose dependent manner (Eijkelkamp et al., 2011). This proposed that unlike common iron chelators, curcumin acts in a manner that is independent of a reduction in cell viability. From the expression profile, we also observed that a number of genes involved in energy production are overexpressed in the presence of curcumin (Figure 8) proposing normal bacterial metabolism. The products of five genes (BPSL1554, BPSL1782, BPSL2469, BPSS0495, BPSS1855) that were induced may compensate for the loss of energy production caused by the three repressed genes (BPSL0247, BPSS0278, BPSS1744). Furthermore, the BPSS0495 gene that encodes a nitroreductase family protein was induced. The induction of this gene suggested that *B. pseudomallei* may use alternative sources such as nitrogen to generate energy. This idea is supported by the up-regulation of BPSS0369 that encodes bacterioferritin ferredoxin. The BPSS0369 gene is located upstream of the bacterioferritin gene (*bfr*) encoding a 64 amino acid-residue protein identical to a region of NifU, a [2Fe-2S] protein found in nitrogen-fixing bacteria (Tuanyok et al., 2005).

## Discussion

Melioidosis has long been associated with high morbidity and mortality in endemic areas and requires protracted long-term antibiotic treatment (Wiersinga et al., 2006). Emergence of antibiotic resistant isolates (Wuthiekanun et al., 2011) demands for an urgent need for new alternative treatments. Therefore, the aim of our study was to search for anti-infectives that specifically target bacterial virulence thus reducing selection pressure toward resistance. Many studies have adopted a similar approach to disrupt pathways related to virulence including inhibiting toxin function, toxin delivery, regulation of virulence expression, bacterial adhesion and biofilm formation (Clatworthy et al., 2007; de la Fuente-Nunez et al., 2013). In general, the *B. pseudomallei* genome content is distributed over 2 chromosomes (chromosomes 1 and 2) that demonstrate significant functional partitioning. Chromosome 1 is associated with core functions such as cell metabolism and growth while chromosome 2 carries more genes required for accessory functions such as adaptation for survival and virulence factors (Holden et al., 2004). Our transcriptome data demonstrated that 62.5% (55 genes) of the differentially regulated genes are located in chromosome 2 whilst 37.5% (33 genes) are in chromosome 1. The data from this study suggests that the anti-infective effect of curcumin targets more accessory functions compared to functions related to cell proliferation.

In our study, we used the *B. pseudomallei* -*C. elegans* infection model to screen a collection of natural products for any that rescued the worms from infection by means of attenuating bacterial virulence. One of these products, curcumin, was able to enhance worm survival without affecting bacterial survival. The approach we adopted involved screening all the natural products or constituent ingredients against the infected worms first, rather than beginning the study with the antimicrobial screen, in an effort to maximize our search for potential anti-infectives. Although some products are deleterious to the bacteria, at sub-MIC concentrations, they may be good anti-infective candidates capable of attenuating virulence factors (Rudrappa and Bais, 2008). Furthermore, the animal model screen would simultaneously allow us to identify products that rescue the worm via modulating the host immune response (Hamill et al., 2008; Easton et al., 2009). When we pre-treated both host and *B. pseudomallei* with curcumin prior to infection, we found that worm survival was enhanced under experimental conditions proposing that curcumin affects both host immunity (data not shown) and bacterial virulence and/or survival (Figure 6).

Curcumin, also known as diferuloylmethane, is a polyphenol derived from turmeric. Many beneficial characteristics of curcumin such as antimicrobial, anticancer, and antioxidant have been documented (Aggarwal et al., 2007). In recent years, curcumin has also attracted interest for its ability to delay aging and age-related diseases (Sikora et al., 2010; Liao et al., 2011). In our study, different biochemical tests demonstrated that curcumin affects *B. pseudomallei* lipase and protease. We also showed that curcumin impedes biofilm formation. Studies have shown that curcumin is anti-virulent toward various pathogens, mainly attributable to its anti-quorum sensing (anti-QS) trait (Rudrappa and Bais, 2008; Packiavathy et al., 2014). Bacterial QS systems regulate virulence especially the secretion of toxins and also biofilm formation (Antunes et al., 2010). We hypothesized that curcumin attenuates *B. pseudomallei* by inhibiting the QS mechanism. We went on to test this hypothesis by measuring the global gene expression in curcumin-treated bacteria.

Unexpectedly, the transcriptome analysis did not reflect the reduction in *B. pseudomallei* secretion of lipase and protease or biofilm formation. Expression of genes corresponding to these virulence factors and those involved in QS were not regulated. This may be a result of analysis on the bacteria at different phases of growth in the various experiments. Whilst the bacteria used in the *C. elegans* survival assays and biochemical tests were treated for 24–48 h, a 10-h log phase curcumin-treated culture was used to harvest the RNA for transcriptome analysis. *B. pseudomallei* is known to express different sets of genes during distinct growth phases (Rodrigues et al., 2006). In our analysis, we found that most of the up-regulated genes encode proteins involved in iron acquisition and transport as well as hypothetical proteins and proteins of unknown function. The iron chelating effect of curcumin most likely created iron-restricted growth conditions which forced the bacteria to increase expression of iron acquisition and transport genes. Nevertheless, a large number of genes induced in the presence of curcumin are annotated as hypothetical and unknown function. Hence, it is possible that these genes could be coding for virulence related factors that contribute to the biofilm phenotype. The up-regulation of several transcriptional regulators also points to potential indirect effects which may directly or indirectly control these hypothetical proteins.

Siderophore has been proposed as a virulence factor of *B. pseudomallei* (Yang et al., 1993). Nevertheless, the induction of siderophore gene expression upon curcumin treatment did not lead to accelerated worm killing. On the other hand, survival of *C. elegans* fed with curcumin-treated bacteria was significantly enhanced when compared to the untreated control (Figure 6). As curcumin chelates iron, this may have affected bacterial fitness, hence, the bacteria switched their focus onto iron acquisition to ensure growth and maintenance rather than infection of the worms. The microarray analysis also showed more genes involved in energy metabolism were induced (Figure 8), supporting our observation that curcumin did not inhibit bacterial growth. Expression of BPSS0495 (nitroreductase family protein) and BPSS0369 (bacterioferritin ferredoxin) suggested that the bacteria were capable of utilizing alternative metabolic pathways for energy production.

In conclusion, we propose that curcumin is a potential anti-infective agent against *B. pseudomallei*. This natural product exerts non-favorable conditions on the bacteria's growth, thus, forcing the bacteria to re-focus on attaining fitness as opposed to killing of the host. Whilst curcumin has been documented as an anti-infective toward various pathogens, this is the first report that demonstrates the ability of curcumin to prolong the lifespan of the *B. pseudomallei* infected host. These preliminary findings provide invaluable insight into the interaction between curcumin and *B. pseudomallei*.

## Author contributions

SE and SN conceived and designed the experiments. SE performed the experiments. SE and SN analyzed the results and wrote the paper.

### Conflict of interest statement

The authors declare that the research was conducted in the absence of any commercial or financial relationships that could be construed as a potential conflict of interest.

## Supplementary material

Supplementary Data 1 **Detailed information of all 42 natural products used in the anti-infective screening**.

Supplementary Data 2 **List of differentially expressed *B. pseudomallei* genes in response to curcumin treatment**.

## References

[B1] Adonizio A. Leal S. M. Jr. Ausubel F. M. Mathee K. (2008). Attenuation of *Pseudomonas aeruginosa* virulence by medicinal plants in a *Caenorhabditi elegans* model system. J. Med. Microbiol. 57, 809–813. 10.1099/jmm.0.47802-0 18566137

[B2] Afzal A. Oriqat G. Khan M. A. Jose J. Afzal M. (2013). Chemistry and biochemistry of terpenoids from *Curcuma* and related species. J. Biol. Active Prod. Nat. 3, 1–55 10.1080/22311866.2013.782757

[B3] Aggarwal B. B. Sundaram C. Malani N. Ivhikawa H. (2007). Curcumin: the Indian solid gold. Adv. Exp. Med. Biol. 595, 1–75. 10.1007/978-0-387-46401-5_1 17569205

[B4] Alice A. F. Lopez C. S. Lowe C. A. Ledesma M. A. Crosa J. H. (2006). Genetic and transcriptional analysis of the siderophore malleobactin biosyntesis and transport genes in the human pathogen *Burkholderia pseudomallei*. J. Bacteriol. 188, 1551–1556. 10.1128/JB.188.4.1551-1566.2006 16452439 PMC1367220

[B5] Ansel H. C. Norred W. P. Roth I. L. (1969). Antimicrobial activity od dimethyl sulfoxide against *Escherichia coli*, *Pseudomonas aeruginosa*, and *Bacillus megaterium*. J. Pharm. Sci. 58, 836–839. 10.1002/jps.2600580708 4980332

[B6] Antunes L. C. Ferreira R. B. Buckner M. M. Finlay B. B. (2010). Quorum sensing in bacterial virulence. Microbiology 156, 2271–2282. 10.1099/mic.0.038794-0 20488878

[B7] Ashdown L. R. Koehler J. M. (1990). Production of hemolysin and other extracellular enzymes by clinical isolates of *Pseudomonas pseudomallei*. J. Clin. Microbiol. 28, 2331–2334. 2229359 10.1128/jcm.28.10.2331-2334.1990 PMC268171

[B8] Baum L. Ng A. (2004). Curcumin interaction with copper and iron suggested one possible mechanism of action in Alzheimer's disease animal models. J. Alzheimers Dis. 6, 367–377. 15345806 10.3233/jad-2004-6403

[B9] Beanan M. J. Strome S. (1992). Characterization of a germ-line proliferation mutation in C. elegans. Development 116, 755–766. 1289064 10.1242/dev.116.3.755

[B11] Cegelski L. Marshall G. R. Eldridge G. R. Hultgren S. J. (2008). The biology and future prospects of antivirulence therapies. Nat. Rev. Microbiol. 6, 17–27. 10.1038/nrmicro1818 18079741 PMC2211378

[B12] Cheng A. C. Currie B. J. (2005). Melioidosis: epidemiology, pathophysiology, and management. Clin. Microbiol. Rev. 18, 383–416. 10.1128/CMR.18.2.383-416.2005 15831829 PMC1082802

[B13] Chieng S. Carreto L. Nathan S. (2012). *Burkholderia pseudomallei* transcriptional adaptation in macrophages. BMC Genomics 13:328. 10.1186/1471-2164-13-328 22823543 PMC3418162

[B14] Choh L. C. Ong G. H. Vellasamy K. M. Kalaiselvam K. Kang W. T. Al-Maleki A. R.. (2013). B*urkholderia* vaccines: are we moving forward? Front. Cell. Infect. Microbiol. 5:5. 10.3389/fcimb.2013.00005 23386999 PMC3564208

[B15] Clatworthy A. E. Pierson E. Hung D. T. (2007). Targeting virulence: a new paradigm for antimicrobial therapy. Nat. Chem. Biol. 3, 541–548. 10.1038/nchembio.2007.24 17710100

[B16] Clinical and Laboratory Standards Institute (CLSI). (2006). Performance Standards for Antimicrobial Disk Susceptibility Test: Approved Standard, 9th Edn CLSI Document M2-A9. Wayne, PA: Clinical and Laboratory Standard Institute.

[B17] Cruz-Migoni A. Hautberque G. M. Artymiuk P. J. Baker P. J. Bokori-Brown M. Chang C. T.. (2011). A *Burkholderia pseudomallei* toxin inhibits helicase activity of translation factor eIF4A. Science 334, 821–824. 10.1126/science.1211915 22076380 PMC3364511

[B18] de la Fuente-Nunez C. Reffuveille F. Fernandez L. Hancock R. E. (2013). Bacterial biofilm development as an multicellular adaptation: antibiotic resistance and new therapeutic strategies. Curr. Opin. Microbiol. 16, 580–589. 10.1016/j.mib.2013.06.013 23880136

[B19] Easton D. M. Nijnik A. Mayer M. L. Hancock R. E. (2009). Potential of immunomodulatory host defense peptides as novel anti-infectives. Trends Biotechnol. 27, 582–590. 10.1016/j.tibtech.2009.07.004 19683819 PMC7114281

[B20] Eijkelkamp B. A. Hassan K. A. Paulsen I. T. Brown M. H. (2011). Investigation of the human pathogen *Acinetobactor baumannii* under iron limiting conditions. BMC Genomics 12:126. 10.1186/1471-2164-12-126 21342532 PMC3055841

[B21] Gan Y. H. Chua K. L. Chua H. H. Liu B. Hii C. S. Chong H. L.. (2002). Characterization of *Burkholderia pseudomallei* infection and identification of novel virulence factors using a *Caenorhabditis elegans* host system. Mol. Microbiol. 44, 1185–1197. 10.1046/j.1365-2958.2002.02957.x 12068805

[B22] Garsin D. A. Sifri C. D. Mylonakis E. Qin X. Singh K. V. Murray B. E.. (2001). A simple model host for identifying Gram-positive virulence factors. Proc. Natl. Acad. Sci. U.S.A. 98, 10892–10897. 10.1073/pnas.191378698 11535834 PMC58570

[B23] Gunes H. Gulen D. Mutlu R. Gumus A. Tas T. Eren Topkaya A. (2013). Antibacterial effects of curcumin: an *in vitro* minimum inhibitory concentration study. Toxicol. Ind. Health. [Epub ahead of print]. 10.1177/0748233713498458 24097361

[B24] Hamill P. Brown K. Jensses H. Hancock R. E. (2008). Novel anti-infectives: is host defense the answer? Curr. Opin. Biotechnol. 19, 628–636. 10.1016/j.copbio.2008.10.006 19000763

[B25] Hara Y. Mohamed R. Nathan S. (2009). Immunogenic *Burkholderia pseudomallei* outer membrane proteins as potential candidate vaccine targets. PLoS ONE 4:e6496. 10.1371/journal.pone.0006496 19654871 PMC2716516

[B26] Harland D. N. Dassa E. Titbal R. W. Brown K. A. Atkins H. S. (2007). ATP-binding cassette systems in *Burkholderia pseudomallei* and *Burkholderia mallei*. BMC Genomics 8:83. 10.1186/1471-2164-8-83 17391530 PMC1853089

[B27] Holden M. T. Titbal R. W. Peacock S. J. Cerdeno-Tarraga A. M. Atkins T. Crossman L. C.. (2004). Genomic plasticity of the causative agent of melioidosis, *Burkholderia pseudomallei*. Proc. Natl. Acad. Sci. U.S.A. 101, 14240–14245. 10.1073/pnas.0403302101 15377794 PMC521101

[B28] Jiao Y. Wilkinson J. IV Di X. Wang W. Hatcher H. Knock N. D.. (2009). Curcumin, a cancer chemopreventive and chemotherapeutic agent, is a biologically active iron chelator. Blood 113, 462–469. 10.1182/blood-2008-05-155952 18815282 PMC2615657

[B29] Jiao Y. Wilkinson J. IV Pietsch C. E. Buss J. L. Wang W. Planalp R.. (2006). Iron chelation in the biological activity of curcumin. Free Radic. Biol. Med. 40, 1152–1160. 10.1016/j.freeradbiomed.2005.11.003 16545682

[B30] Koh S. F. Tay S. T. Puthucheary S. D. (2013). Colonial morphology and biofilm forming ability of *Burkholderia pseudomallei*. Trop. Biomed. 30, 428–433. 24189672

[B31] Kong C. Tan M. W. Nathan S. (2014b). *Orthosiphon stamineus* protects *Caenorhabditis elegans* against *Staphylococcus aureus* infection through immunomodulation. Biol. Open 3, 644–655. 10.1186/1472-6882-14-4 24972867 PMC4154301

[B32] Kong C. Yehye W. A. Rahman N. A. Tan M. W. Nathan S. (2014a). Discovery of potential anti-infectives against *Staphylococcus aureus* using a *Caenorhabditis elegans* infection model. BMC Complement. Altern. Med. 14:4. 10.1242/bio.20148334 24393217 PMC3893568

[B33] Kouker G. Jaeger K. E. (1987). Specific and sensitive plate assay for bacterial lipases. Appl. Environ. Microbiol. 53, 211–213. 3103532 10.1128/aem.53.1.211-213.1987 PMC203632

[B34] Krewulak K. D. Vogel H. J. (2008). Structural biology of bacterial iron uptake. Biochim. Biophys. Acta 1778, 1781–1804. 10.1016/j.bbamem.2007.07.026 17916327

[B35] Kumarasamy K. K. Toleman M. A. Walsh T. R. Bagaria J. Butt F. Balakrishnan R.. (2010). Emergence of a new antibiotic resistance mechanism in India, Pakistan, and the UK: a molecular, biological and epidemiological study. Lancet Infect. Dis. 10, 597–602. 10.1016/S1473-3099(10)70143-2 20705517 PMC2933358

[B36] Lee S. H. Chong C. E. Lim B. S. Chai S. J. Sam K. K. Mohamed R. (2007). *Burkholderia pseudomallei* animal and human isolates from Malaysia exhibit different phenotypic characteristics. Diagn. Microbiol. Dis. 58, 263–270 10.1016/j.diagmicrobio.2007.01.002 17350202

[B36a] Lee S. H. Ooi S. K. Mahadi N. M. Tan M. W. Nathan S. (2011). Complete killing of *Caenorhabditis elegans* by *Burkholderia pseudomallei* is dependent on prolonged direct association with the viable pathogen. PLoS ONE 6:16707 10.1371/journal.pone.0016707 PMC3049773 21408228

[B37] Lee S. H. Wong R. R. Chin C. Y. Lim T. Y. Eng S. A. Kong C.. (2013). *Burkholderia pseudomallei* suppresses *Caenorhabditis elegans* immunity by specific degradation of a GATA transcription factor. Proc. Natl. Acad. Sci. U.S.A. 110, 15067–15072. 10.1073/pnas.1311725110 23980181 PMC3773767

[B38a] Liao V. H. Yu C. W. Chu Y. J. Li W. H. Hsieh Y. C. Wang T. T. (2011). Curcumin-mediated lifespan extension in *Caenorhabditis elegans*. Mech. Ageing Dev. 132, 480–487. 10.1016/j.mad.2011.07.008 21855561

[B38] Li S. Yuan W. Deng G. Wang P. Aggarwal B. B. (2011). Chemical composition and product quality control of turmeric (*Curcuma longa* L.). Pharm. Crops 2, 28–54 10.2174/2210290601102010028

[B39] Limmathurosatkul D. Wongratanacheewin S. Teerawattanasook N. Wongsuvan G. Chaisuknant S. Chetchotisakd P.. (2010). Increasing incidence of human melioidosis in northeast Thailand. Am. J. Trop. Med. Hyg. 82, 1113–1117. 10.4269/ajtmh.2010.10-0038 20519609 PMC2877420

[B40] Marathe S. A. Ray S. Chakravortty D. (2010). Curcumin increases the pathogenicity of *Salmonella enterica* serovar Typhimurium in murine model. PLoS ONE 5:e11511. 10.1371/journal.pone.0011511 20634977 PMC2901387

[B41] Moghadamtousi S. Z. Kadir H. A. Hassandarvish P. Tajik H. Abubakar S. Zandi K. (2014). A review on antibacterial, antiviral, and antifungal activity of curcumin. Biomed. Res. Int. 2014:186864. 10.1155/2014/186864 24877064 PMC4022204

[B42] Moy T. I. Ball A. R. Anklesaria Z. Casadei G. Lewis K. Ausubel F. M. (2006). Identification of novel antimicrobials using a live-animal model system. Proc. Natl. Acad. Sci. U.S.A. 103, 10414–10419. 10.1073/pnas.0604055103 16801562 PMC1482800

[B43] Neelakantan P. Subbarao C. Sharma S. Subbarao C. V. Garcia-Godoy F. Gutman J. L. (2013). Effectiveness of curcumin against *Enterococcus faecalis* biofilm. Acta. Odontol. Scand. 71, 1453–1457. 10.3109/00016357.2013.769627 23394209

[B44a] Newman D. J. Cragg G. M. (2012). Natural products as sources of new drugs over the 30 years from 1981 to 2010. J. Nat. Prod. 75, 311–335. 10.1021/np200906s 22316239 PMC3721181

[B44] Nijnik A. (2013). Immunomodulatory approaches for prevention and treatment of infectious diseases. Curr. Opin. Microbiol. 16, 590–595. 10.1016/j.mib.2013.06.011 23870826

[B45] Ooi S. K. Lim T. Y. Lee S. H. Nathan S. (2012). *Burkholderia pseudomallei* kills *Caenorhabditis elegans* through virulence mechanism distinct from intestinal lumen colonization. Virulence 3, 485–496. 10.4161/viru.21808 23076282 PMC3524147

[B46] O'Quinn A. L. Wiegand E. M. Jeddeloh J. A. (2001). *Burkholderia pseudomallei* kills the nematode *Caenorhabditis elegans* using an endotoxin-mediated paralysis. Cell Microbiol. 3, 381–393. 10.1046/j.1462-5822.2001.00118.x 11422081

[B47] Packiavathy I. A. Priya S. Pandian S. K. Ravi A. V. (2014). Inhibition of biofilm development of uropathogens by curcumin - an anti-quorum sensing agent from *Curcuma longa*. Food Chem. 148, 453–460. 10.1016/j.foodchem.2012.08.002 24262582

[B48] Papa R. Artini M. Cellini A. Tilotta M. Galano E. Pucci P.. (2013). A new anti-infective strategy to reduce the spreading of antibiotic resistance by the action on adhesion-mediated virulence factors in *Staphylococcus aureus*. Microb. Pathog. 63, 44–53. 10.1016/j.micpath.2013.05.003 23811076

[B49] Patel N. Conojero L. De Reynal M. Easton A. Bancroft G. J. Titball R. W. (2011). Development of vaccines against *Burkholderia pseudomallei*. Front. Microbiol. 2:198. 10.3389/fmicb.2011.00198 21991263 PMC3180847

[B50] Puah S. M. Puthucheary S. D. Wang J. T. Pan Y. J. Chua K. H. (2014). Molecular characterization of putative virulence determinants in *Burkholderia pseudomallei*. ScientificWorldJournal 2014:590803. 10.1155/2014/590803 25215325 PMC4158159

[B52] Puthucheary S. D. Sam I. C. (2012). Why is the response rate slow in ceftazidime therapy for melioidosis? Expert Rev. Anti. Infect. Ther. 10, 5–7. 10.1586/eri.11.158 22149608

[B53] Rodrigues F. Sarkar-Tyson M. Harding S. V. Sim S. H. Chua H. H. Lin C. H.. (2006). Global map of growth-regulated gene expression in *Burkholderia pseudomallei*, the causative agent of melioidosis. J. Bacteriol. 188, 8178–8188. 10.1128/JB.01006-06 16997946 PMC1698202

[B54] Rudrappa T. Bais H. P. (2008). Curcumin, a known phenolic from *Curcuma longa*, attenuates the virulence of *Pseudomonas aeruginosa* PA01 in whole plant and animal pathogenicity models. J. Agric. Food Chem. 56, 1955–1962. 10.1021/jf072591j 18284200

[B55] Sarabhai S. Sharma P. Capalash N. (2013). Ellagic acid derivatives from *Terminalia chebula* Retz. downregulate the expression of quorum sensing genes to attenuate *Pseudomonas aeruginosa* PAO1 virulence. PLoS ONE 8:e53441. 10.1371/journal.pone.0053441 23320085 PMC3539995

[B56] Schwyn B. Neilands J. B. (1987). Universal chemical assay for the detection and determination of siderophores. Anal. Biochem. 160, 47–56. 10.1016/0003-2697(87)90612-9 2952030

[B57] Shapira M. Tan M. W. (2008). Genetic analyis of *Caenorhabditis elegans* innate immunity. Methods Mol. Biol. 415, 429–442. 10.1007/978-1-59745-570-1_25 18370169

[B58] Sikora E. Scapagini G. Barbagallo M. (2010). Curcumin, inflammation, ageing and age-related diseases. Immun. Ageing 17:1 10.1186/1742-4933-7-1 20205886 PMC2827376

[B59] Silva B. S. Dow S. W. (2013). Development of *Burkholderia mallei* and *pseudomallei* vaccines. Front. Cell Infect. Microbiol. 3:10. 10.3389/fcimb.2013.00010 23508691 PMC3598006

[B60] Tabara H. Hills R. J. Mello C. C. Priess J. R. Kohara Y. (1999). *pos-1* encodes a cytoplasmic zinc-finger protein essential for germline specification in *Caenorhabditis elegans*. Development 126, 1–11. 9834181 10.1242/dev.126.1.1

[B61] Tan M. W. Mahajan-Miklos S. Ausubel F. M. (1999). Killing of *Caenorhabditis elegans* by *Pseudomonas aeruginosa* used to model mammalian bacterial pathogenesis. Proc. Natl. Acad. Proc. Sci. U.S.A. 96, 715–720 10.1073/pnas.96.2.715 PMC15202 9892699

[B62] Tuanyok A. Kim H. S. Nierman W. C. Yu Y. Dunbar J. Moore R. A.. (2005). Genome-wide expression of iron regulation in *Burkholderia pseudomallei* and *Burkholderia mallei* using DNA microarray. FEMS Microbiol. Lett. 252, 327–335. 10.1016/j.femsle.2005.09.043 16242861

[B63] Wiegand I. Hilpert K. Hancock R. E. W. (2008). Agar and broth dilution method to determine the minimal inhibitory concentration (MIC) of antimicrobial substances. Nat. Protoc. 3, 163–175. 10.1038/nprot.2007.521 18274517

[B64] Wiersinga W. J. van der Poll T. White N. J. Day N. P. Peacock S. J. (2006). Melioidosis: insights into the pathogenicity of *Burkholderia pseudomallei*. Nat. Rev. Microbiol. 4, 272–282. 10.1038/nrmicro1385 16541135

[B65] Wuthiekanun V. Amornchai P. Saoprom N. Chantratita N. Chierakul W. Koh G. C. K. W.. (2011). Survey of antimicrobial resistance in clinical *Burkholderia pseudomallei* isolates over two decades in northeast Thailand. Antimicrob. Agents Chemother. 55, 5388–5391. 10.1128/AAC.05517-11 21876049 PMC3195054

[B66] Yang H. Kooi C. D. Sokol P. A. (1993). Ability of *Pseudomonas pseudomallei* malleobactin to acquire transferrin-bound, lactoferrin-bound, and cell-derived iron. Infect. Immun. 61, 656–662. 7678587 10.1128/iai.61.2.656-662.1993 PMC302777

[B67] Zilberberg M. D. Shorr A. F. (2013). Prevalence of multidrug-resistant *Pseudomonas aeruginosa* and carbapenem-resistant *Enterobacteriaceae* among specimens from hospitalized patients with pneumonia and bloodstream infections in the United States from 2000 to 2009. J. Hosp. Med. 8, 559–563. 10.1002/jhm.2080 24022878

